# Advanced gastric cancer with metachronous intracranial oligometastases without recurrence after multidisciplinary team discussion and comprehensive treatment: a case report

**DOI:** 10.3389/fonc.2023.1268199

**Published:** 2023-11-03

**Authors:** Lijuan Shao, Hongyu Zhang, Wenting He, Jun Wu, Danxia Zhu, Haibo Cheng

**Affiliations:** ^1^ Department of Oncology, The Third Affiliated Hospital of Soochow University, Changzhou, China; ^2^ The First Clinical Medical College, Nanjing University of Chinese Medicine, Nanjing, China; ^3^ Jiangsu Collaborative Innovation Center of Traditional Chinese Medicine Prevention and Treatment of Tumor, Nanjing, China

**Keywords:** gastric adenocarcinoma, pembrolizumab, oligometastasis, metastasectomy, neuroendocrine differentiation, multidisciplinary team

## Abstract

This article describes the process of multidisciplinary team (MDT) discussion and comprehensive treatment of a case of advanced gastric cancer that tested positive for programmed death ligand 1 (PD-L1). During diagnosis, the patient presented with advanced gastric cancer and numerous unresectable metastases in the lesser omental lymph nodes, both lungs, liver, and left parietal occipital lobe. A meeting was arranged for the departments of oncology, gastrointestinal surgery, radiotherapy, imaging, and pathology to discuss the case. Initially, the patient had a partial response to the first-line treatment, which was a combination of pembrolizumab and chemotherapy. However, after nineteen months, the patient presented with a metachronous isolated lesion in the left frontal lobe. After mutual agreement among the oncology, brain surgery, gastrointestinal surgery, radiotherapy, imaging, and pathology departments, the intracranial lesion underwent resection. Following this, the operation was supplemented by stereotactic radiation therapy (SRT) and whole-brain radiation therapy (WBRT). The patient showed excellent signs of recovery after the operation, and her general condition remained favorable after 16 months of follow-up. Nonetheless, the outlook for patients facing advanced-stage gastric cancer remains distressing. Through multidisciplinary team (MDT) discussions, patients diagnosed with advanced gastric cancer can receive standardized diagnostic and treatment approaches to develop reasonable and personalized comprehensive treatment plans. Such plans help to improve the quality of life of patients and effectively prolong their survival time.

## Introduction

Gastric cancer is a frequently occurring malignant tumor of the digestive tract that is the fourth most common cancer-related cause of death globally (1). Adenocarcinoma is the most common pathological type of gastric cancer, characterized mainly by distant metastases. Gastric cancer often metastasizes to the local lymph nodes, liver, lung, bone and peritoneum. Ovarian metastasis is sometimes observed in women. However, brain metastasis occurs less often, with a 6.5% incidence rate (2). Current treatment options for brain metastases in gastric cancer consist of surgical resection, whole-brain radiotherapy, stereotactic radiotherapy and systemic chemotherapy.

Currently, the multidisciplinary comprehensive treatment mode for tumors has become the standard mode of modern tumor treatment. Survival time of gastric cancer patients can be extended, especially in advanced and complex patients, through extensive discussions, individual patient assessment, medical records analysis, implementation of ideal treatment strategies, and the combination of local and systemic treatment concepts.

Metachronous oligometastatic disease (OMD) in the brain caused by advanced gastric cancer is rare. In the present case, a patient with advanced gastric cancer underwent multidisciplinary management with a standardized diagnosis and treatment. As of now, the patient has survived for over three years without any cancer recurrence or metastasis.

## Case report

**Figure 1:**
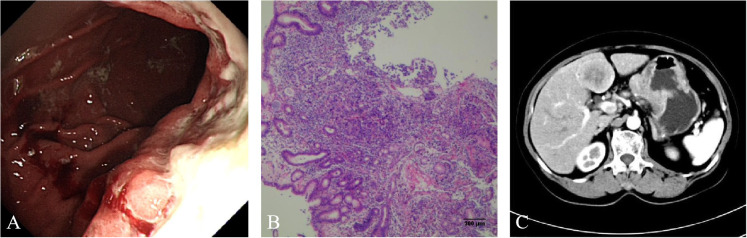
**(A)** Gastroscopy showed that irregular giant ulcers in the cardia with a peripheral dike, which was covered with dirty moss on the surface. **(B)** Hematoxylin and eosin staining suggested adenocarcinoma. **(C)** Computed tomography (CT) of the upper abdomen showed thickening of the gastric wall in the cardia and the lesser curvature of the stomach, enlargement of lymph nodes in the lesser omentum and space occupying lesions of liver.

**Figure 2:**
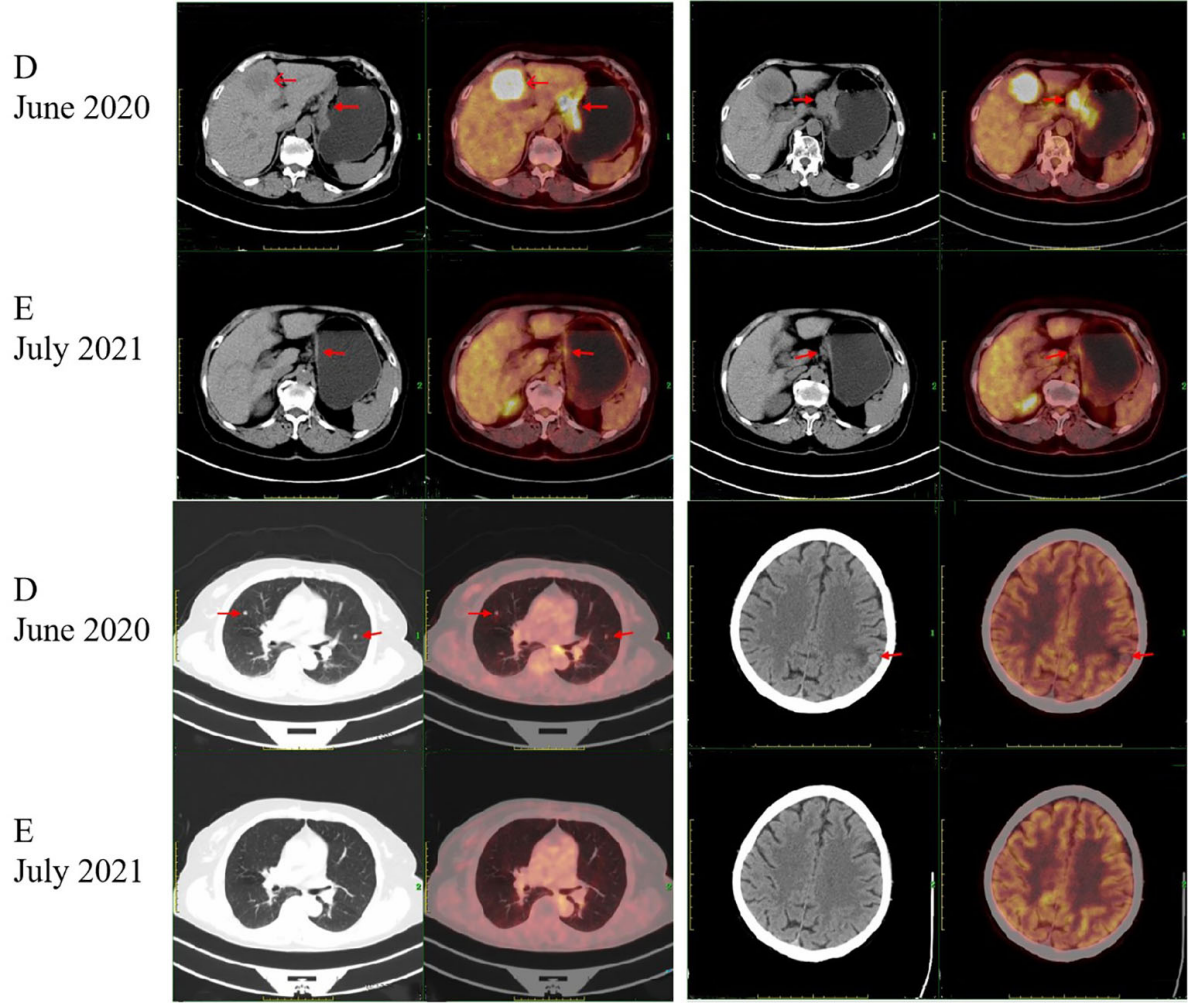
Positron emission tomography/computed tomography (PET/CT) images. Arrows indicate tumors. **(D)** PET/CT scan showed intense accumulation of the (18) F-fluorodeoxyglucose (FDG) metabolism at the cardia and the lesser curvature of the stomach, the gastric lesser omental lymph nodes, both lungs, liver and left parietal occipital lobe at the time of initial diagnosis. **(E)** After the HYPERLINK "javascript:;" treatment of pembrolizumab plus chemotherapy and pembrolizumab monotherapy, PET/CT scan showed that the lesions in the cardia and the lesser curvature of the stomach and the gastric lesser omental lymph nodes were significantly reduced and the metabolic activity was reduced. The lesions in both lungs, liver and left parietal occipital lobe disappeared.

A 75-year-old Chinese woman complained of dull pain and discomfort in the upper abdomen in June 2020. The patient had a history of high blood pressure, type 2 diabetes, and tuberculosis. She underwent gastroscopy, which revealed irregular giant ulcers in the cardia with a peripheral dike covered with dirty moss on the surface (**Figure 1A**). The histology report indicated adenocarcinoma in the cardia (**Figure 1B**). Immunohistochemistry revealed positivity for MSH6, MSH2, MLH1, and PMS2, with a PD-L1 composite positive score (CPS) of approximately 10 and CerbB2 negativity. The upper abdominal CT scan revealed the thickening of the gastric wall in the cardia and lesser curvature of the stomach, enlarged lymph nodes in the lesser omentum, and liver with space-occupying lesions (See **Figure 1C**). The PET/CT illustrated high accumulations of (18)F-fluorodeoxyglucose (FDG) metabolism in the cardia and lesser curvature of the stomach, gastric lesser omental lymph nodes, both lungs, liver and left parietal occipital lobe (**Figure 2D**).

There was a MDT discussion between the oncology, gastrointestinal surgery, radiotherapy, imaging and pathology departments. Based on the expert opinion of the MDT, the patient was diagnosed with gastric cancer with metastases in the lesser omental lymph nodes, both lungs, the liver and the left parietal occipital lobe, for which resection was not feasible. Considering the patient’s positive immunohistochemistry for PD-L1 and negative for HER-2, the recommended course of action was immunotherapy combined with chemotherapy as the first-line treatment. The patient should be monitored vigilantly for adverse reactions related to immunotherapy medication and chemotherapy during the course of treatment. Following the MDT discussion, the patient was prescribed pembrolizumab (PD-1 inhibitor) in addition to chemotherapy. Starting from July 2020, the patient underwent three cycles of S-1 and oxaliplatin (SOX) treatment. Following the treatment, the patient experienced bone marrow suppression and underwent seven cycles of chemotherapy with a revised regimen of fluorouracil, leucovorin, and oxaliplatin (FOLFOX). Between July 2020 and April 2021, the patient was administered Pembrolizumab for nine cycles at the same time. The patient, due to an apparent oral ulcer and an incapability to withstand intravenous chemotherapy, was given pembrolizumab monotherapy for 14 cycles beginning from April 2021 and ending in February 2022. During this period, the patient underwent regular CT scans of the whole body. The CT scans showed a significant decrease in the thickening and strengthening of the stomach wall at the cardia and the lesser curvature of the stomach. The lesions in the lesser omental lymph nodes decreased gradually. Concurrently, the lesions located in the lungs, liver, and left parietal occipital lobe demonstrated significant reduction up until complete resolution. The PET/CT scan performed in July 2021 showed a significant reduction in lesions in the cardia and lesser curvature of the stomach, as well as in the gastric lesser omental lymph nodes, compared to the results of the PET/CT scan performed in June 2020. There was also a decrease in metabolic activity. Lesions in the lungs, liver and left parietal occipital lobe disappeared (**Figure 2E**). Consequently, the patient showed a partial response.

**Figure 3:**
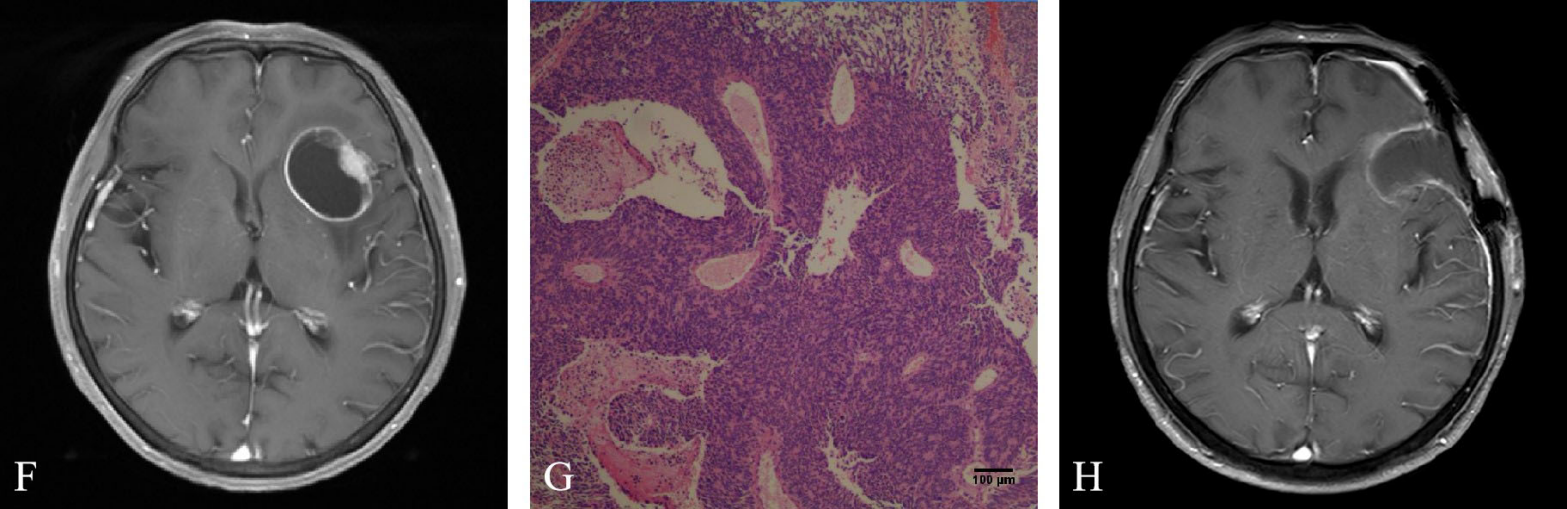
**(F)** Brain magnetic resonance imaging (MRI) showed a lesion in the left frontal lobe. **(G)** Hematoxylin and eosin staining suggested metastatic poorly differentiated neuroendocrine carcinoma. **(H)** Brain MRI showed enhanced edge and adjacent meninges after brain tumor surgery.

In January 2022, the patient developed vertigo. Magnetic resonance imaging (MRI) of the brain showed a lesion in the left frontal lobe (**Figure 3F**). Comparison of PET/CT imaging in January 2022 and PET/CT imaging in July 2021 showed that the lesions in the cardia and lesser curvature of the stomach and the gastric lesser omental lymph nodes were similar to those before (the lesions had mild metabolic activity). The low density nodule in the left frontal lobe with slightly increased FDG uptake was relatively new. In February 2022, the patient experienced severe vertigo. Brain MRI showed that the left frontal lobe was taking up more space than before (January 2022).

**Figure 4:**
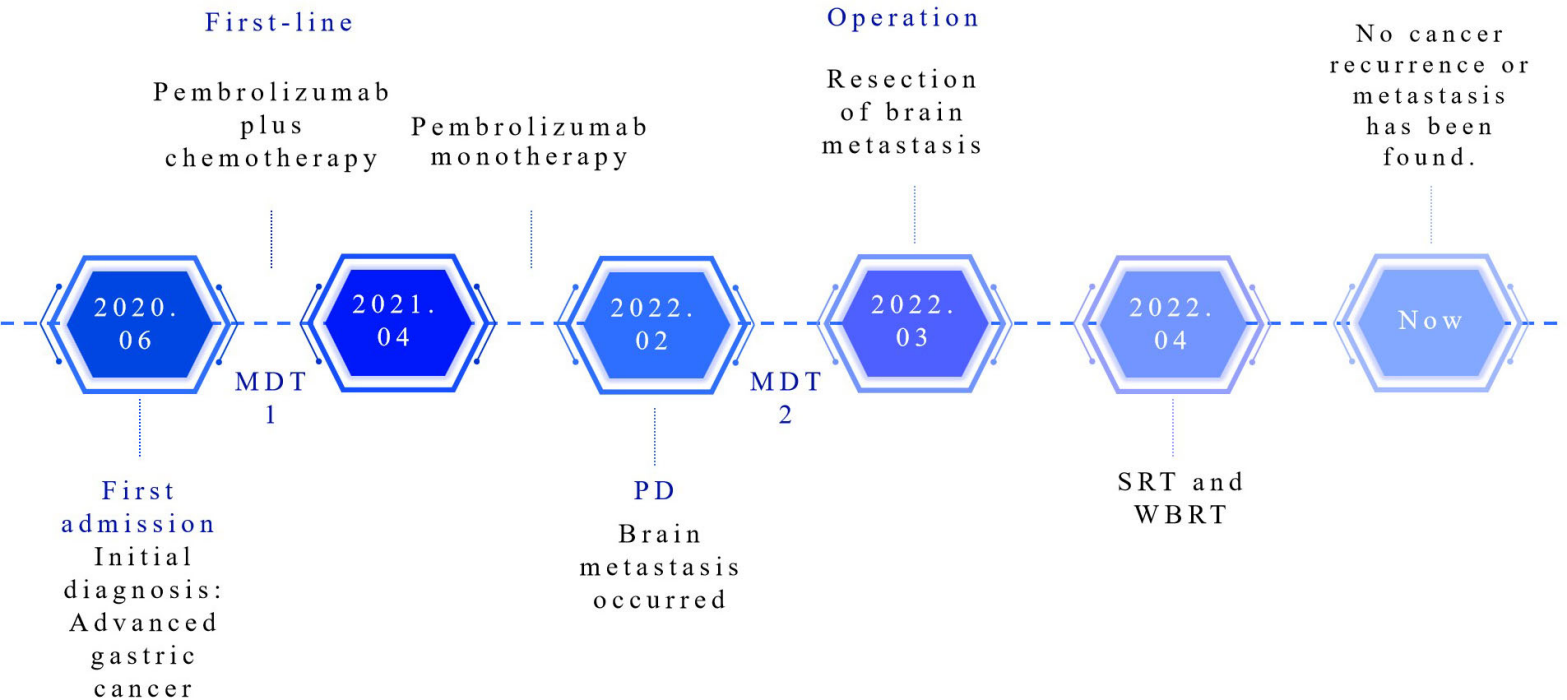
Treatment process flow chart for this case. MDT, multidisciplinary team; PD, progressive disease; SRT, stereotactic radiation therapy; WBRT, whole brain radiation therapy.

After MDT discussion by the departments of oncology, brain surgery, gastrointestinal surgery, radiotherapy, imaging and pathology, the main opinions were the following: After systemic treatment, the tumor was controlled in other parts of the body. The lesion in the left frontal lobe was a metachronous OMD located in the safe area of the brain. The patients were generally in good condition and resection of the brain metastases was feasible. The patient’s family members were informed of the intraoperative findings, possible adverse outcomes, and the advantages and disadvantages of different surgical approaches. After obtaining their consent and signing the informed consent form, resection of the brain metastasis was performed in March 2022. Histology revealed metastatic poorly differentiated neuroendocrine carcinoma in the left frontal lobe (**Figure 3G**). Immunohistochemistry showed that AE1/AE3, CK7, Villin, Syn, MSH6, MSH2, MLH1 and PMS2 were positive, CerbB2, CK20, CDX-2 (-), CgA, Vimentin, GFAP, EMA, TTF-1 were negative, Ki67 was more than 60% and PD-L1 (CPS) was about 5. Brain MRI in April 2022 showed enhancement of the rim and adjacent meninges after brain tumor surgery (**Figure 3H**). Therefore, the patient underwent SRT and WBRT. At present, the patient’s general condition was good. A flow chart of the treatment process is shown in **Figure 4**.

## Discussion

In this case, the gastric cancer was found to have metastasized throughout the patient’s body at the time of initial diagnosis. Surgery was not considered necessary or appropriate. In cases of multiple systemic metastases of gastric cancer, the primary systemic treatments are chemotherapy consisting of fluoropyrimidine and platinum. For patients who test positive for HER-2, trastuzumab targeted therapy can also be administered. The pattern of systemic treatment for advanced gastric cancer has been changed by immunotherapy, represented by immune checkpoint inhibitors, which can improve the prognosis and quality of life of patients. Advancement of line number has become the first-line standard treatment over time. Pembrolizumab is an IGG4-κ monoclonal antibody that selectively binds with high affinity to PD-1 and its ligands (PD-L1 and PD-L2) (3), thereby improving T-cell-mediated antitumor effects. In patients with metastatic PD-L1-positive gastric cancer (4), Pembrolizumab exhibited a controllable toxic profile and promising antitumor activity. In patients with PD-L1-positive, HER2-negative advanced gastric or gastroesophageal junction cancer (5), chemotherapy in combination with pembrolizumab showed a favorable response rate and a controlled safety profile in KEYNOTE-659. The objective response rate (ORR) and median progression-free survival (PFS) were 72.2% and 9.4 months respectively. Advanced gastric cancer with microsatellite stability (MSS) type, CerbB2 negative and PD-L1 positive was detected in this patient. The first-line treatment selected for the patient was the combination of chemotherapy S-1 and oxaliplatin (SOX) with pembrolizumab. As a result of these treatments, the patient experienced myelosuppression and was switched to FOLFOX. In later treatment, pembrolizumab was used alone. These treatments demonstrated good clinical efficacy for the patient. Regarding adverse reactions, the patient experienced grade 2 weakness, grade 2 vomiting, and grade 4 myelosuppression during immunotherapy combined with chemotherapy, but these adverse reactions improved after symptomatic treatment or adjustment of medication.

Reports indicate that combining PD-1 inhibitor with chemotherapy as a first-line treatment can improve the survival of patients with advanced gastric cancer. The CheckMate 649 test confirmed that nivolumab (a PD-1 inhibitor) combined with chemotherapy as the first-line treatment for unresectable, untreated advanced gastric, gastro-esophageal junction, and oesophageal adenocarcinoma can significantly improve the overall survival (OS) and progression-free survival (PFS) of patients with a PD-L1 CPS≥5 compared to those who receive only chemotherapy. There were also significant benefits in OS that were observed in patients with a PD-L1 CPS≥1 and in the fully randomized population (6). Hence, the approval of nivolumab combined with chemotherapy as a first-line treatment covers all patients, providing a new treatment alternative for patients with more advanced gastric cancer. ORIENT-16 is a phase III randomized double-blind study of Sintilimab (a PD-1 inhibitor) combined with chemotherapy as a first-line treatment for advanced gastric cancer (7). The study demonstrated significant overall survival (OS) benefits in both CPS≥5 population and the general population. This study was the first to achieve an increase in OS benefit to 15.2 months in the general population. Therefore, immunotherapy should be administered in the primary stage and as early as possible to augment patient’s survival. Continued advances in immunotherapy will further enhance the tumor microenvironment of patients with potential surgical opportunities and potentially alter the treatment modalities for conversion therapy and neoadjuvant therapy.

The patient developed vertigo after 19 months of treatment. A new lesion was observed in the brain on combined plain and enhanced MRI. In recent years, the significance of MDT discussions in formulating treatment plans, evaluating surgical indications, defining safe and feasible surgical plans and scheduling postoperative follow-up has been increasingly recognized. Gastric cancer can lead to a serious complication known as brain metastasis (BRM). Early detection of brain metastases (BRM) was found to be a prognostic factor. Patients who had BRM successfully resected had a better prognosis than those with unresectable BRM (2). Local treatment of oligometastatic disease (OMD) using stereotactic body radiotherapy (SBRT) or metastatic resection, along with systemic therapy, improves overall survival (OS) compared to systemic therapy alone (8). In this case, it was observed that extensive metastasis of gastric cancer resulted in the formation of oligometastasis in the brain after systemic treatment. A MDT discussed treatment strategies for the intracranial lesion. The neurological surgery team and radiation oncologists determined the surgical treatment for the intracranial lesion.

Following resection of the brain tumor, the specimen showed the presence of neuroendocrine carcinoma (NEC). In this case, the patient had a non-functional NEC, as no abnormalities of the endocrine system were found. NEC can manifest in nearly any organ, with the lungs and digestive system being the most common (9). Intracranial NEC may be categorized as primary or metastatic. Primary intracranial NEC is notably uncommon (10, 11). Intracranial NEC in the clinical context is usually metastatic, with the majority of cases occurring due to lung lesions. Intracranial metastatic NEC comprises of 1.5% to 5% of all brain metastatic tumors. In most cases, the patients with this condition have already developed local or distant metastases in other parts of the body (12). Therefore, in this case, we need to first consider whether the intracranial lesion is a metastatic tumor (13) and check for any potential lesion sites in other areas of the body. CT and PET/CT scans revealed no lesions in the lungs and other body parts, apart from the lesions present in the cardia and the lesser curvature of the stomach, and the omental lymph nodes of the stomach. Therefore, we considered that the primary focus of intracranial NEC in this case was gastric adenocarcinoma. It is worth noting that prior to the administration of chemotherapy, there was no evidence of neuroendocrine carcinoma in the gastric lesion tissue. Tumors are actually heterogeneous. Chemotherapy-induced cytotoxic injury during gastric cancer growth may inhibit the growth of adenocarcinoma cells. Daughter cells exhibit molecular biological or genetic changes following multiple divisions and proliferation, which results in the formation of neuroendocrine tumor cells. The resistance of neuroendocrine tumor cells to cytotoxic damage may be responsible for the spread of brain neuroendocrine tumors (14, 15). According to Warraich (16), a case of gastric adenocarcinoma transformed into a neuroendocrine tumor due to a Sister Mary Joseph Nodule. For an intracranial solitary tumor causing obvious space-occupying effects, it is essential to remove the tumor as thoroughly as possible while preserving brain function, regardless of whether the tumor is primary or metastatic. It may be necessary to expand the resection appropriately for a solitary lesion in a non-functional region, followed by postoperative adjuvant radiotherapy and chemotherapy. The patient experienced chest tightness and shortness of breath following radiotherapy, which could not be definitively attributed to immunotherapy, but the symptoms improved after receiving corticosteroid treatment. The patient was given oral etoposide chemotherapy intermittently due to respiratory symptoms. The patients’ tumor markers were closely monitored and the level of carcinoembryonic antigen was found to be elevated at the time of initial diagnosis and remained normal after treatment. There was no observed increase in the level of neuron-specific enolase.

Brain CT or MRI should be routinely performed for patients with gastric cancer to assess their condition at the time of diagnosis and throughout the treatment process. The early detection of brain metastases from gastric cancer, along with timely recourse to local and systemic treatments, can be helpful in improving the prognosis of patients. Effective discussions by MDT (Multidisciplinary Team) can assist patients in making adequate and considered treatment decisions. Furthermore, it enables them to customize the selection of immunotherapy, chemotherapy, surgery and radiotherapy regimens so that patients with advanced gastric cancer can gain significant benefits such as improvements in quality of life and increased survival periods.

## Data availability statement

The original contributions presented in the study are included in the article/supplementary material, Further inquiries can be directed to the corresponding author.

## Ethics statement

The studies involving humans were approved by the ethics committee of Soochow University. The studies were conducted in accordance with the local legislation and institutional requirements. The participants provided their written informed consent to participate in this study. Written informed consent was obtained from the individual(s) for the publication of any potentially identifiable images or data included in this article.

## Author contributions

LS: Data curation, Funding acquisition, Writing – original draft, Writing – review & editing. HZ: Data curation, Investigation, Writing – review & editing. WH: Data curation, Investigation, Writing – review & editing, Funding acquisition. JW: Investigation, Project administration, Supervision, Writing – review & editing. DZ: Data curation, Funding acquisition, Writing – review & editing. HC: Conceptualization, Data curation, Formal Analysis, Writing – review & editing.

## References

[1] Sung H Ferlay J Siegel RL Laversanne M Soerjomataram I Jemal A. Global Cancer Statistics 2020: GLOBOCAN estimates of incidence and mortality worldwide for 36 cancers in 185 countries. CA Cancer J Clin (2021) 71:209–49. doi: 10.3322/caac.21660 33538338

[2] Brunner M Soll D Adler K Sasse A König U Mekolli A. Brain metastases in gastroesophageal cancers-an underestimated complication. Gastric Cancer (2022) 25:161–9. doi: 10.1007/s10120-021-01219-z PMC8732847 34297239

[3] Chung HC Bang YJ Fuchs CS Qin SK Satoh T Shitara K. First-line pembrolizumab/placebo plus trastuzumab and chemotherapy in HER2-positive advanced gastric cancer: KEYNOTE-811. Future Oncol (2021) 17:491–501. doi: 10.2217/fon-2020-0737 33167735 PMC8411394

[4] Muro K Chung HC Shankaran V Geva R Catenacci D Gupta S. Pembrolizumab for patients with PD-L1-positive advanced gastric cancer (KEYNOTE-012): a multicentre, open-label, phase 1b trial. Lancet Oncol (2016) 17:717–26. doi: 10.1016/S1470-2045(16)00175-3 27157491

[5] Kawazoe A Yamaguchi K Yasui H Negoro Y Azuma M Amagai K. Safety and efficacy of pembrolizumab in combination with S-1 plus oxaliplatin as a first-line treatment in patients with advanced gastric/gastroesophageal junction cancer: Cohort 1 data from the KEYNOTE-659 phase IIb study. Eur J Cancer (2020) 129:97–106. doi: 10.1016/j.ejca.2020.02.002 32145474

[6] Janjigian YY Shitara K Moehler M Garrido M Salman P Shen L. First-line nivolumab plus chemotherapy versus chemotherapy alone for advanced gastric, gastro-oesophageal junction, and oesophageal adenocarcinoma (CheckMate 649): A randomised, open-label, phase 3 trial. Lancet (2021) 398:27–40. doi: 10.1016/S0140-6736(21)00797-2 34102137 PMC8436782

[7] Xu J Jiang H Pan Y Gu K Cang S Han L. LBA53 Sintilimab plus chemotherapy (chemo) versus chemo as first-line treatment for advanced gastric or gastroesophageal junction (G/GEJ) adenocarcinoma (ORIENT-16): First results of a randomized, double-blind, phase III study. Ann Oncol (2021) 32:S1331. doi: 10.1016/j.annonc.2021.08.2133

[8] Kroese TE Christ SM van Rossum PSN Burger MDL Buijs GS Mühlematter U. Incidence and survival of patients with oligometastatic esophagogastric cancer: A multicenter cohort study. Radiother Oncol (2022) 173:269–76. doi: 10.1016/j.radonc.2022.06.012 35753555

[9] Baxi AJ Chintapalli K Katkar A Restrepo CS Betancourt SL Sunnapwar A. Multimodality imaging findings in carcinoid tumors: A head-to-toe spectrum. Radiographics (2017) 37:516–36. doi: 10.1148/rg.2017160113 28287937

[10] Hallet J Law CH Cukier M Saskin R Liu N Singh S. Exploring the rising incidence of neuroendocrine tumors: a population-based analysis of epidemiology, metastatic presentation, and outcomes. Cancer (2015) 121:589–97. doi: 10.1002/cncr.29099 25312765

[11] Nakaguchi H Matsuno A Miyawaki S Murakami M Yamada M Yamazaki K. Small cell carcinoma originating from the cavernous sinus. Acta Neurochir (Wien) (2010) 152:493–500. doi: 10.1007/s00701-009-0389-z 19434364

[12] Kuroda N Inenaga C Arai Y Otsuki Y Tanaka T. Cerebellar metastasis of unknown primary neuroendocrine carcinoma: report of case mimicking hemangioblastoma. World Neurosurg (2019) 128:320–23. doi: 10.1016/j.wneu.2019.05.104 31125774

[13] Lobins R Floyd J. Small cell carcinoma of unknown primary. Semin Oncol (2007) 34:39–42. doi: 10.1053/j.seminoncol.2006.10.027 17270664

[14] Shia J Tickoo SK Guillem JG Qin J Nissan A Hoos A. Increased endocrine cells in treated rectal adenocarcinomas: a possible reflection of endocrine differentiation in tumor cells induced by chemotherapy and radiotherapy. Am J Surg Pathol (2002) 26:863–72. doi: 10.1097/00000478-200207000-00004 12131153

[15] Oneda E Liserre B Bianchi D Rota L Savelli G Zorzi F. Diagnosis of Mixed Adenoneuroendocrine Carcinoma (MANEC) after neoadjuvant chemotherapy for pancreatic and gastric adenocarcinoma: two case reports and a review of the literature. Case Rep Oncol (2019) 12:434–42. doi: 10.1159/000501200 PMC6600038 31275134

[16] Warraich MS Fidai S Gandhi S. An umbilical mass in a young female. Gastroenterology (2022) 163:382–83. doi: 10.1053/j.gastro.2022.04.033 35489429

